# Colorectal endoscopic submucosal dissection using a gas-free saline-immersion dissection technique

**DOI:** 10.1055/a-2155-6107

**Published:** 2023-09-15

**Authors:** Tatsuma Nomura, Shinya Sugimoto, Yoshikazu Hayashi, Ryutaro Matsushima, Jun Oyamada, Keiichi Ito, Tomonori Yano

**Affiliations:** 1 Department of Gastroenterology, Ise Red Cross Hospital, Ise, Japan; 2 Department of Gastroenterology, Mie Prefectural Shima Hospital, Shima, Japan; 3 Department of Medicine, Division of Gastroenterology, Jichi Medical University, Shimotsuke, Japan

Recently, a new technique for performing underwater endoscopic submucosal dissection (ESD) in a saline solution has been reported 1 2 . Although using a tapered hood is considered beneficial for underwater ESD in saline immersion, the bubbles generated during ESD can hinder smooth dissection 3 . We describe a gas-free saline-immersion technique for ESD, in which a tapered hood is filled with saline by pressing an air/water valve button, thus moving the bubbles to outside the hood.

First, the hole for carbon dioxide (CO _2_ ) in the bottle cap is closed using a neoprene rubber plug (ARAM, Osaka, Japan) ( Fig. 1 ). Subsequently, when the air/water valve button is pressed, the saline solution, but not the CO _2_ , is infused. Filling the hood with the saline solution pushes out the bubbles generated during ESD, thus maintaining an optimal endoscopic view.

**Fig. 1:**
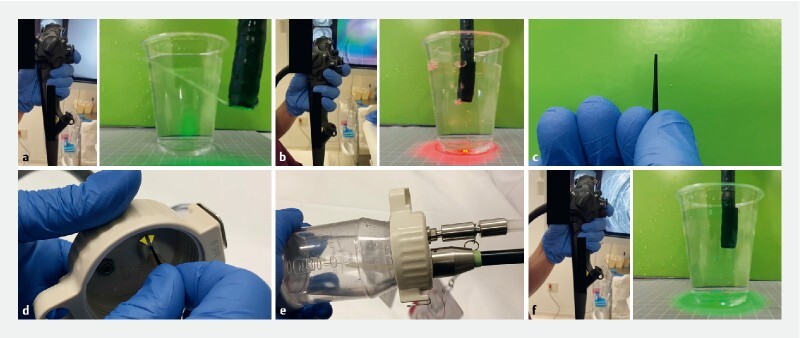
Solution infusion without carbon dioxide (CO _2_ ) insufflation. **a** Firmly pressing the air/water valve button enables water infusion. **b** Before the air/water valve button is pressed firmly, the CO _2_ present in the endoscope is pushed out, which obstructs the endoscopic field of vision. **c** Plug used to prevent CO _2_ gas insufflation. **d** The plug is inserted into the bottle’s CO _2_ outlet (yellow arrowheads). **e** The bottle is filled with saline instead of water. **f** Pressing the button firmly enables saline infusion without CO _2_ insufflation.

The patient in the case presented here had a 30-mm sessile serrated lesion overlying a diverticulum in the sigmoid colon ( Fig. 2 ). We performed ESD with en bloc resection of the SSL using a calibrated, small-caliber-tip, transparent hood to access the submucosa within the diverticulum 4 ( Video 1 ). Although the submucosa remains visible during saline immersion, bubbles are generated when the submucosa is dissected, which obstruct the endoscopic view. Therefore, a gas-free saline-immersion technique was used to dissect the submucosa while pressing the air/water valve button with the index finger. By pressing and holding the button, the saline is infused into the space inside the hood, which removes the bubbles produced by ESD. This continuous saline infusion facilitates smooth dissection by preventing the obstruction of the endoscopic field of vision by gas. Furthermore, the saline immersion technique softens the mucosa and muscles, enabling easy closure of the defect using the reopenable clip-over-line method 5 .

**Fig. 2:**
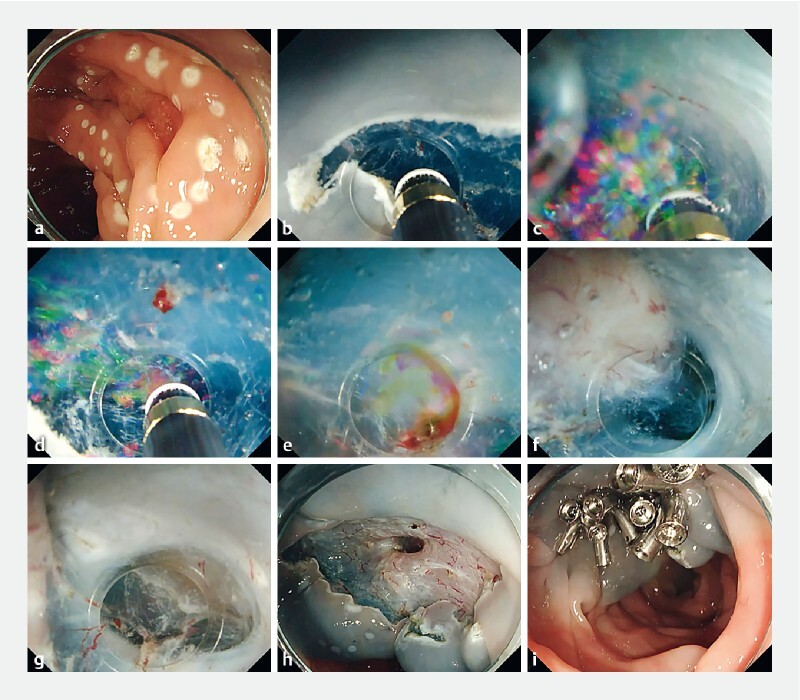
Gas-free saline-immersion technique for endoscopic submucosal dissection (ESD). **a** A 30-mm sessile serrated lesion (SSL) completely overlying a diverticulum in the sigmoid colon. **b** Mucosal incision. **c** Poor endoscopic visibility due to the bubbles passing into the hood during ESD under saline immersion. **d** When the air/water valve button is pressed and the submucosal layer is dissected while the saline is being pumped, the pressure of the saline prevents bubbles from entering the hood. **e** When bleeding occurs, the pressure of the saline makes it difficult for blood to enter the tapered hood. **f, g** Dissection of the submucosa within the diverticulum. **h** Mucosal and muscle layer defects within the diverticulum following ESD. **i** The mucosal defect has been completely closed using the reopenable clip-over-line method (ROLM).

**Video 1** A gas-free saline-immersion technique, using a plug that allows saline infusion without carbon dioxide (CO _2_ ) gas insufflation, was used to perform endoscopic submucosal dissection (ESD) of a sessile serrated lesion (SSL) in the sigmoid colon.


The gas-free saline-immersion technique for ESD is a feasible method with no CO _2_ gas emission during saline immersion.

Endoscopy_UCTN_Code_TTT_1AQ_2AD

## Notes

Endoscopy E-Videos

https://eref.thieme.de/e-videos
*E-Videos* is an open access online section of the journal *Endoscopy* , reporting on interesting cases and new techniques in gastroenterological endoscopy. All papers include a high-quality video and are published with a Creative Commons CC-BY license. Endoscopy E-Videos qualify for HINARI discounts and waivers and eligibility is automatically checked during the submission process. We grant 100% waivers to articles whose corresponding authors are based in Group A countries and 50% waivers to those who are based in Group B countries as classified by Research4Life (see: https://www.research4life.org/access/eligibility/ ). This section has its own submission website at https://mc.manuscriptcentral.com/e-videos

## References

[1] Yahagi N Nishizawa T Sasaki M Water pressure method for duodenal endoscopic submucosal dissection Endoscopy 2017 49 E227 E228 28759932 10.1055/s-0043-113556

[2] Nagata M Usefulness of underwater endoscopic submucosal dissection in saline solution with a monopolar knife for colorectal tumors (with videos) Gastrointest Endosc 2018 87 1345 1353 29242059 10.1016/j.gie.2017.11.032

[3] Nagata M Tapered hood with wide holes in its sides for efficient air-bubble removal during underwater endoscopic submucosal dissection Dig Endosc 2022 34 654 35000224 10.1111/den.14232

[4] Nomura T Sugimoto S Oyamada J GI endoscopic submucosal dissection using a calibrated, small-caliber-tip, transparent hood for lesions with fibrosis VideoGIE 2021 6 301 304 34278091 10.1016/j.vgie.2021.03.001 PMC8267961

[5] Nomura T Sugimoto S Temma T Mucosal defect closure using a calibrated, small-caliber-tip, transparent hood after colorectal endoscopic submucosal dissection Endoscopy 2022 54 E691 E692 35180793 10.1055/a-1750-8800

